# Pathologic analysis of non-neoplastic parenchyma in renal cell carcinoma: a comprehensive observation in radical nephrectomy specimens

**DOI:** 10.1186/s12885-017-3849-5

**Published:** 2017-12-28

**Authors:** Xun Wang, Qiang Liu, Wen Kong, Jiwei Huang, Yonghui Chen, Yiran Huang, Jin Zhang

**Affiliations:** 1 Department of Urology Renji Hospital affiliated to Shanghai Jiaotong, University School of Medicine, No.1630, Dongfang Road, Shanghai, 200127 China; 2 Department of Pathology Renji Hospital affiliated to Shanghai Jiaotong, University School of Medicine, No.1630, Dongfang Road, Shanghai, 200127 China

**Keywords:** Renal cell carcinoma, Non-neoplastic parenchyma, Histological changes, Pseudo-capsule, Compressed band

## Abstract

**Background:**

This study provides a comprehensive examination of the histological features of non-neoplastic parenchyma in renal cell carcinoma (RCC). We prospectively collected radical nephrectomy (RN) specimens, to analyze the histological changes within peritumoral and distant parenchyma.

**Methods:**

Data of patients who underwent RN and had no known history of diabetes, hypertension, hyperlipidemia, or chronic kidney disease etc., were prospectively collected. Tumor pseudo-capsule (PC), and parenchyma within 2 cm from tumor margin, were pathologically assessed. The parenchyma beyond PC or tumor margin was divided into 20 subsections of 1 mm in width. Histological changes, including chronic inflammation, glomerulosclerosis, arteriosclerosis and nephrosclerosis, were given scores of 0, 1, 2 or 3 for each subsection of each specimen, according to their severity. The 20 subsections of each specimen were further divided into four groups according to the distance from the tumor edge (group 1: 0–2 mm; group 2: 2–5 mm; group 3: 5–10 mm; group 4: 10–20 mm), to better compare the peritumoral parenchyma with the distant parenchyma.

**Results:**

In total, 53 patients were involved in this study. All tumors were confirmed RCCs (clear cell vs. papillary vs. chromophobe were 83% vs. 5.7% vs. 11.3%, respectively), with a mean size of 5.6 cm. Histological changes were more severe in peritumoral parenchyma close to PC or tumor edge (0–5 mm), and less common within parenchyma more distant from the tumor (5–20 mm) (*p* < 0.001). chronic inflammation and nephrosclerosis were the most common changes especially in peritumoral parenchyma (0-2 mm). PC was present in 49 tumors (92.5%), and PC invasion occurred in 5 cases (10.2%). Mean PC thickness was 0.7 mm. PCs were more likely to be present in clear cell RCC or papillary RCC than in chromophobe RCC (100% vs. 100% vs. 33.3%, respectively; p < 0.001).

**Conclusions:**

Most RCCs have a well-developed PC, especially clear cell RCC. Histological changes mainly occur in peritumoral parenchyma, being rather uncommon in distant parenchyma. A compression band filled with severe histological changes was typically observed in renal parenchyma close to the tumor. Its preservation while performing an enucleation margin may not be entirely necessary.

**Electronic supplementary material:**

The online version of this article (10.1186/s12885-017-3849-5) contains supplementary material, which is available to authorized users.

## Background

A limited number of studies have focused on the comprehensive pathological analysis of non-neoplastic parenchyma in renal cell carcinoma (RCC), which includes the peritumoral parenchyma and the more distant parenchyma from the tumor. Former research [1–7] has only focused on peritumoral tissues, showing that various histological changes occur in the non-neoplastic parenchyma in RCC, such as chronic inflammation (CI), glomerulosclerosis (GS) or arteriosclerosis (AS). Recent reports [8–10] have begun to analyze the areas of renal tissues located further from the tumor, proving that the peritumoral tissue cannot represent the condition of the entire non-neoplastic parenchyma. However, most of these studies involved partial nephrectomy (PN) specimens. As a result, the pathological observations were limited to the tissue very close to the lesion. To fully understand the histological changes of the renal parenchyma in RCC, peritumoral parenchyma and distant parenchyma, as well as the pattern of tumor invasion within the renal parenchyma, should be more thoroughly examined.

Our study exclusively enrolled radical nephrectomy (RN) specimens to better assess the non-neoplastic parenchyma, including the tumor pseudo-capsule (PC), peritumoral parenchyma and distant parenchyma.

## Methods

This single-center prospective study was approved by the institutional review board, and the requirement for informed consent was waived. We prospectively enrolled 53 patients, who met the following eligibility criteria: patients with typical Enhanced Computed Tomography or Magnetic Resonance Imaging images indicating a single RCC lesion; patients undergoing RN at our institution from August 2015 to May 2016; pathological confirmation of RCC in surgical specimens; patients without medical conditions potentially affecting the renal parenchyma, including hypertension, hyperlipidemia, diabetes mellitus or chronic kidney disease etc. Patients who did not eventually undergo RN or who had pathological diagnosis other than RCC were further excluded from the study.

We prospectively recorded the basic information of the patients, including age, gender, and surgical approach (open, laparoscopic, robotic). The size of the primary tumor, histologic subtype, Fuhrman grade, pathological TNM stage and margin status were recorded on regular pathological examination, including hematoxylin-eosin and immunohistochemical stains. Tumor features were evaluated according to the 2004 World Health Organization (WHO) classification [11], the Fuhrman grading system [12], and the 2010 American Joint Committee on Cancer TNM staging. [13]

Apart from the regular pathological examination, each specimen was further sampled to include the PC of the tumor (if present), as well as renal parenchyma of at least 2 cm in width from around the PC or tumor margin (if PC was absent). Three to four hematoxylin-eosin slides were made per case, in addition to the standard sections that were used for clinical assessment.

All slides were reviewed by two urological pathologists (QL and JX) blinded to the patients’ clinical parameters and tumor pathological information, including age, gender, surgical approach, tumor size and subtype. For each specimen, information on PC, including the presence of PC, PC thickness, as well as the presence of tumor invasion within PC, were recorded. The 2-cm-wide renal parenchyma specimens from beyond the PC or tumor margin were further divided into 20 subsections of 1 mm in width. The histological changes of parenchyma, including CI, GS, AS and NS, were graded in each subsection of each specimen, according to the criteria shown in Table 1. CI, GS, AS and NS were scored on the histological scale of severity (Additional file 1 Figure S1-S4). These criteria have previously been used by other authors for similar purposes [8, 14]. A minimum of three random microscopic fields were required to evaluate the histological changes in each subsection. The highest grade observed in the three fields was recorded. A dividing optical microscope was used for the measurement of length.

**Table 1:** Grading criteria for each histologic change

	Grade 0	Grade 1	Grade 2	Grade 3
CI	no	<3 lymphoid aggregates	> = 3 lymphoid aggregates	diffuse inflammatory cell infiltrate
GS	no	<25% glomeruli sclerosed	25–50%	>50%
AS	no	both vessels wall thickening and luminal narrowing were slight	partial vessels luminal occlusion <= 50%	vessels luminal occlusion >50%
NS	no	thickened tubular basement membranes and hyaline sclerosis surrounding the tubules when examined at 20X	the grade1 changes along with interstitial fibrosis could be observed at 4X	diffuse tubular atrophy and drop-out with extensive hyaline sclerosis observed at 4X

CI, chronic inflammation, was graded by using random objective 40X microscopic field; GS, glomerulosclerosis, was graded by using 10X objective field; AS, arteriosclerosis, was graded by using 40X field; NS, nephrosclerosis;

In order to better compare the peritumoral parenchyma with the distant parenchyma, we grouped the 20 subsections into four groups of variable widths, according to their distance from the PC or tumor edge, as follows: group 1: 0–2 mm; group 2: 2–5 mm; group 3: 5–10 mm and group 4: 10–20 mm. Finding the histological change mentioned before in any subsection of the group was defined as positive occurrence. The frequency of each change among the four groups was recorded. The parenchyma score for each histological change was calculated by averaging all subsection scores in the four groups, to represent the overall severity of the lesion.

One-way ANOVA was used to compare the means of histologic grade and histologic score, among renal parenchyma with different distances from PC or tumor margin. Pearson’s chi-square or Fisher’s exact test were used to compare the occurrence rate of PC in tumors with various characteristics (histologic subtype, Fuhrman grade, pT classification). Student’s t test or one-way ANOVA were used to compare the thickness of PC. All tests were two-sided, and *p*-value <0.05 was considered statistically significant. All statistical analyses were performed using SPSS, version 21.0 (IBM, Armonk, NY).

## Results

Following an initial assessment, 55 patients were enrolled in our study. All patients underwent RN and had no known medical history of hypertension, diabetes mellitus or chronic kidney disease. Two patients were diagnosed as upper tract urothelial carcinoma in postoperative pathological examination and were thus excluded from our study. The remaining 53 patients were confirmed to be RCCs (prevalence of clear cell vs. papillary vs. chromophobe was 83% vs. 5.7% vs. 11.3%, respectively). Median tumor size was 5.6 cm (range: 2.0–12.3 cm) and all patients had negative surgical margins. A total of 162 slides were prepared from 53 surgical specimens. The baseline clinical and pathological characteristics of the 53 patients are shown in Table 2.

**Table 2:** Baseline clinical and pathological characteristics of entire 53 patients

Patient Demographics:	All cases, *n* = 53
Mean age (yrs) (SD)	62 (9.8)
No. male gender (%)	30(56.6)
No. right kidney (%)	25(47.2)
Surgical method (%)	
Open	8(15.1)
Laparoscopic	44(83.0)
Robotic	1(1.9)
Tumor Characteristics:	
Mean Size(cm)(SD)	5.6(2.3)
Histologic Subtype (%)	
Clear cell	44(83.0)
Papillary	3(5.7)
Chromophobe	6(11.3)
Fuhrman grade (%)	
1	6(11.3)
2	34(64.2)
3	11(20.8)
4	2(3.8)
pT classification (%)	
1a	19(35.9)
1b	16(30.2)
2a	16(30.2)
2b	1(1.9)
3a	1(1.9)
Negative Margin (%)	53(100)

**Fig. 1:**
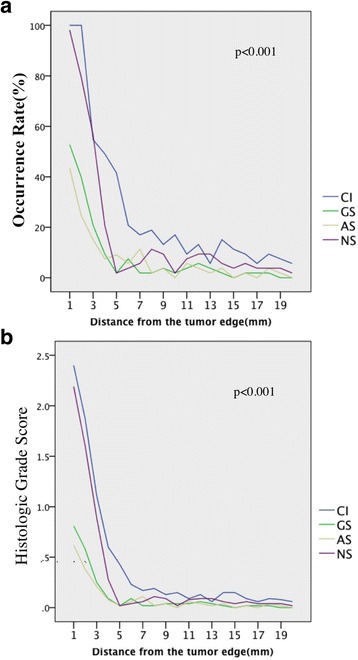
The occurrence rate and histologic grade score correlate with the distance from the tumor edge. **a**, occurrence rate of CI, GS, AS and NS; **b**, histologic grade score of CI, GS, AS and NS

The degree and occurrence rate of all histological changes (CI, GS, AS and NS) decreased with increasing distance from tumor edge or PC (Fig. 1). The nearest group (0–2 mm) had the most severe histological changes. CI and NS were the most common changes in non-neoplastic renal parenchyma, especially in peritumoral parenchyma close to PC or tumor edge.

The 20 subsections were further divided into four groups, as previously defined. Among the four groups, the frequency and severity of histological changes decreased as the distance from PC or tumor edge increased (*P* < 0.001, Table 3). All four histological changes were statistically more common in groups 1 and 2 than in groups 3 and 4 (P < 0.001). However, no significant difference was observed between group 3 and group 4.

**Table 3:** Histologic assessment of non-neoplastic parenchyma in four groups divided according to the distance from the PC or tumor margin

	GROUP 1 (0-2 mm) ***		GROUP 2 (2-5 mm)		GROUP 3 (5-10 mm)		GROUP 4 (10-20 mm)		
	No. (%)*	Grade**	No. (%)	Grade	No. (%)	Grade	No. (%)	Grade	*P* value
CI	53(100)	2.1 (0.6)	29(54.7)	0.72(0.7)	14 (26.4)	0.2(0.2)	8 (15.1)	0.1 (0.13)	<0.001
GS	28(52.8)	0.7 (0.9)	11(20.8)	0.1(0.3)	7 (13.2)	0.09(0.2)	4 (7.5)	0.1(0.1)	<0.001
AS	23(43.4)	0.5 (0.8)	8(15.1)	0.1 (0.3)	5 (9.4)	0.1(0.1)	3 (5.7)	0.1 (0.7)	<0.001
NS	52(98.1)	1.90(0.8)	30(56.6)	0.4 (0.4)	3 (5.7)	0.1(1.0)	2 (3.8)	0.1(0.2)	<0.001

For all 53 specimens, we grouped the 20 subsections (1 mm wide) into four intervals of variable widths according to their distance from the PC or tumor edge. Finding the histological change in any subsection of the group was defined as positive occurrence. The frequency of each change among the four groups was recorded. The grade score of each histologic change was calculated by averaging all subsection scores in the four groups to represent the overall severity of lesion

*Number and precentage of specimens that presents the corresponding histologic change;

**Average grade score and standard deviation of all 53 specimens;

***The distant from pseudo-capsule or tumor margin

PC was present in 49 tumors (92.5%) and its median thickness was 0.7 mm (range: 0.1–4.5 mm). Tumor invasion was observed in 5 PCs: one patient with papillary RCC had tumor invasion across the full thickness of PC, but tumor was not found beyond PC, and the other 4 cases had partial invasion of PC. PCs were more prevalent in clear cell RCC or papillary RCC than in chromophobe RCC (100% vs. 100% vs. 33.3%, respectively *p* < 0.001), and in smaller tumors (≤ 7 cm) than in bigger tumors (> 7 cm) (100% vs. 77.8%, *p* = 0.004). PC invasion was more common in tumors with high nuclear grade (Fuhrman 3–4) (*p* = 0.023), whereas invasion did not correlate with pT classification or histological subtype. PC thickness did not correlate with any tumor pathological characteristics (Table 4).

**Table 4:** Characteristics of the tumor pseudo-capsule (PC) in RCC

	N	PC present(%)	PC invasion(%)	Mean Thickness (mm) (SD)
Histologic Subtype				
Clear cell	44	44(100)	4(9.1)	0.8(0.7)
Papillary	3	3(100)	1(33.3)	0.6(0.3)
Chromophobe	6	2(33.3)	0(0)	0.7(0.2)
Fuhrman grade				
1–2	42	38(90.5)	2(5.3)	0.7(0.3)
3–4	11	11(100)	3(27.3)	0.9(1.2)
pT classification				
pT1	35	35(100)	3(8.6)	0.7(0.3)
1a	19	19(100)	3(15.8)	
1b	16	16(100)	0(0)	
≥ pT2	18	14(77.8)	2(11.1)	1.0(1.1)
2a	16	12(75)	1(8.3)	
2b	1	1(100)	0(0)	
3a	1	1(100)	1(100)	
Overall	53	49(92.5)	5(10.2)	0.7(0.6)

## Discussion

In recent years, emerging data proved that certain histological changes can be identified in the peritumoral parenchyma of RCC [1–7]. In 2013, Garcia-Roig et al. [4] conducted an observational study on 45 patients who underwent PN surgery and who had no known chronic underlying disease. AS was observed in the peritumoral parenchyma of nine patients (20.0%), whereas NS was found in eight patients (17.8%). Gorin et al. [5] observed AS in the peritumoral parenchyma of 29 out of 114 (25.4%) patients with RCC, following PN. These studies suggested that lesions in the peritumoral parenchyma were indicative of subclinical kidney disease and advocated that patients with RCC could potentially benefit from intensive lifestyle modification and medical therapy with lipid-lowering medications [5]. However, more recent studies [8–10] have analyzed broader areas of non-neoplastic renal tissue. Azhar et al.​ [8]. histologically assessed the renal parenchyma in the 0–5 mm range from the tumor edge, and concluded that most histological changes occur in the parenchyma immediately adjacent to the tumor. Kheemes et al. [9] recorded the peritumoral glomerular viability in successive 0.25 cm increments (range 0 to 1 cm), and the mean viable glomeruli positively correlated with the distance from the tumor edge. Furthermore, the authors suggested that glomerular viability near the tumor did not correlate with the preoperative estimated glomerular filtration rate. Our findings agree with these reports: the peritumoral parenchyma is different from the tissue further from the tumor, and the adjacent parenchyma may not reflect the renal function. The data in our study showed that the degree and occurrence rate of histological changes in the parenchyma decreased with increasing distance from the tumor margin or PC.

To comprehensively examine the global non-neoplastic parenchyma, we extended the observation field and utilized RN specimens to compare the peritumoral parenchyma with tissue further from the tumor. In our study, the mean size of the tumors was 5.6 cm (range: 2.0–12.3 cm), and 35% of all tumors were staged T1a. But most of these small tumors were completely endophytic and very close to the renal pedicle. The final surgical decision was made by the surgeons (based on tumor complexity or patients’ will). Because 65% tumors were bigger than 4 cm, and due to the dominant endophytic features of most tumors, many cases could not generate renal tissue beyond 2 cm, despite being RN specimens. Therefore, for consistency, we selected 2 cm peritumoral parenchyma for analysis, in all cases. Obtained results indicated that the 10 mm to 20 mm area had fewer histological changes and seemed to be healthy renal tissue. As the severity of all histological changes decreased with increasing distance from tumor edge or PC, we suggest that this range (1–2 cm) can adequately represent the distant parenchyma.

We found that the peritumoral parenchyma (1–2 mm) was affected by severe inflammation, GS, diffuse tubular atrophy and interstitial fibrosis. These histological changes were far less common in the distant parenchyma (6–20 mm). This may result from the tumor growth and its long-term compression on the peritumoral parenchyma [8]. The renal parenchyma around the tumor was under sustained compression, leading to tiny arterial wall thickening, luminal narrowing and even occlusion. Long-term ischemia and inflammatory cell infiltration within the peritumoral parenchyma result in the appearance of severe histological changes. Moreover, considering the extremely complex mechanism of tumor biology, it remains to be elucidated whether the paracrine effect of tumor cells has a role in this process.

We artificially divided the non-neoplastic parenchyma into 4 groups, according to the distance from the tumor. As a result, a typical narrow area within the peritumoral tissues, filled with high-grade histological changes, was observed in all the specimens, and was named compression band (CB) (Additional file 1: Figure S5). However, no tumor invasion was observed in the CB. As the CB contains few functional glomeruli, preserving all parenchyma adjacent to the renal tumor when deciding the enucleation margin may be not necessary [15]. Azhar and Kheemes’ data [8, 9] also support our hypothesis. Although, traditionally, preserving as much renal parenchyma as feasible while ensuring a negative margin in partial nephrectomy, is a core aim of the surgeons [16–18].

Tumor PC, as a rim of hyperplastic connective tissue, can be found immediately adjacent to the edge of the tumor [19]. PCs are not merely a protection against tumor invasion, but also offer a favorable surgical division in enucleation. Several studies reported on the characteristics of PC [8, 19–21]. Azhar et al. [8] retrospectively observed 124 renal tumors, uncovering PCs in 96% of the total number. Mean PC thickness was 0.7 mm and PC invasion was found in 29% of the tumors. Our data are mainly in agreement with Azhar’s study, though PC invasion only occurred in 10.2% of cases in our study. This result may be due to different tumor characteristics and sampling approaches: all specimens were obtained via RN and mean size was 5.6 cm in our study; while in Azhar’s study, 10% of tumors were benign, 81% were PN specimens and mean size of tumors was 3.9 cm. Apart from the differences amongst the tumors per se, the chosen surgical procedure may also play an important role in those variant results. PN is usually performed along the margins of PC, which may exert destructive and squeezing effects on PCs, because of the intraoperative manipulation. In contrast, RN generally preserves the integrated anatomical structures of the whole specimen. Lastly, our data, as well as that of other studies [8, 20], suggested that tumor PC was more likely presence in clear cell RCCs.

There are several limitations to our study. First, this is a single-institution study, with insufficient sample size. Second, limited cases of papillary or chromophobe subtypes were enrolled in this study, which makes it difficult to draw a definitive conclusion in these subtypes. As the study is an observational research, the variables of renal function were not considered. We will continue the follow-up of these patients in a further study. Yet, we believe these data give a comprehensive description of histological changes in non-neoplastic parenchyma in RCC.

## Conclusions

In agreement with prior reports, our findings suggest that most RCCs have a well-developed pseudo-capsule, especially the clear cell RCC. Histological changes are more common in the peritumoral parenchyma (1–2 mm), whereas the distant parenchyma (10–20 mm) is approaches normal histology. A band of compression with abundant histological changes was generally observed in parenchyma adjacent to PC or tumor margin. Due to few functional glomeruli being present in the parenchyma within the CB, its preservation while performing an enucleation margin may not be entirely necessary.

## Additional file

Additional file 1: Figure S1. (a-d): Chronic inflammation (CI) in non-neoplastic parenchyma by using random objective microscopic fields: A -(40x), grade 0; B- (40x), grade 1; C- (40x), grade 2; D (40x), grade 3.**Figure S2** (a-d): Glomerulosclerosis (GS) in non-neoplastic parenchyma: A -(40x), a single GS change; B- (10x), grade 1; C- (10x), grade 2; D -(10x), grade 3. **Figure S3** (a-d): Arteriosclerosis (AS) in non-neoplastic parenchyma: A- (40x), grade 0; B -(40x), grade 1; C- (40x), grade 2; D- (20x), grade 3. **Figure S4** (a-d): Nephrosclerosis(AS) in non-neoplastic parenchyma. A- (20x), grade 0; B- (20x), grade 1; C- (4x), grade 2; D- (4x), grade 3. **Figure S5**(AB): peri-tumoral parenchyma (1-5 mm) consist of tumor, pseudo-capsule(PC), compressed band(CB) and normal parenchyma . (DOCX 4468 kb)

## Abbreviations

AS: arteriosclerosis
CB: compressed band
CI: chronic inflammation
GS: glomerulosclerosis
NS: nephrosclerosis
PC: pseudo-capsule
PN: partial nephrectomy
RCC: renal cell carcinoma
RN: radical nephrectomy
