# Supplementary material for: Pathologic analysis of non-neoplastic parenchyma in renal cell carcinoma: a comprehensive observation in radical nephrectomy specimens
Source: BMC Cancer. 2017 Dec 28;17:900. doi: 10.1186/s12885-017-3849-5 (PMC5745993; doi:10.1186/s12885-017-3849-5)
Supplement: Additional file 1: Figure S1. — (a-d): Chronic inflammation (CI) in non-neoplastic parenchyma by using random objective microscopic fields: A -(40x), grade 0; B- (40x), grade 1; C- (40x), grade 2; D (40x), grade 3.Figure S2 (a-d): Glomerulosclerosis (GS) in non-neoplastic parenchyma: A -(40x), a single GS change; B- (10x), grade 1; C- (10x), grade 2; D -(10x), grade 3. Figure S3 (a-d): Arteriosclerosis (AS) in non-neoplastic parenchyma: A- (40x), grade 0; B -(40x), grade 1; C- (40x), grade 2; D- (20x), grade 3. Figure S4 (a-d): Nephrosclerosis(AS) in non-neoplastic parenchyma. A- (20x), grade 0; B- (20x), grade 1; C- (4x), grade 2; D- (4x), grade 3. Figure S5(AB): peri-tumoral parenchyma (1-5 mm) consist of tumor, pseudo-capsule(PC), compressed band(CB) and normal parenchyma . (DOCX 4468 kb) [file 12885_2017_3849_MOESM1_ESM.docx]

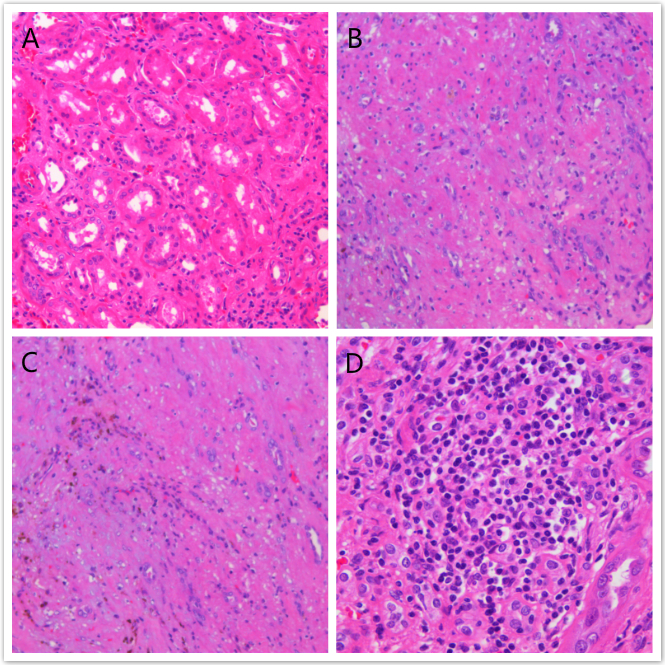


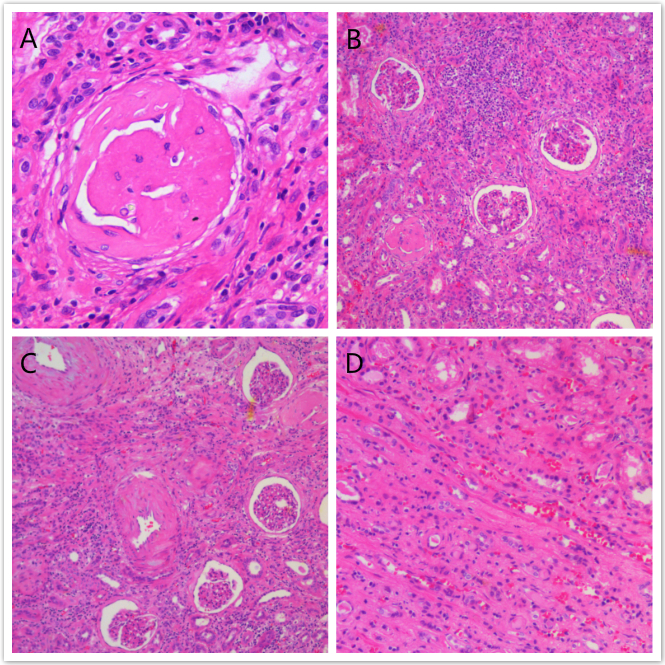


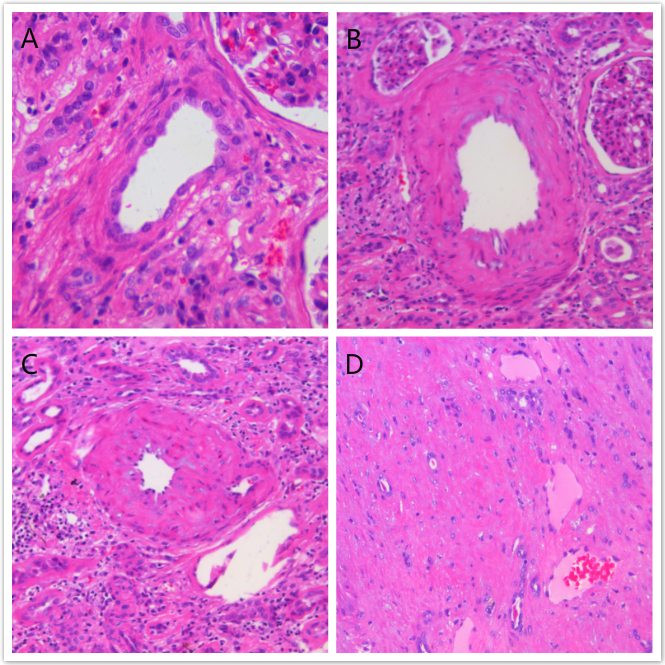


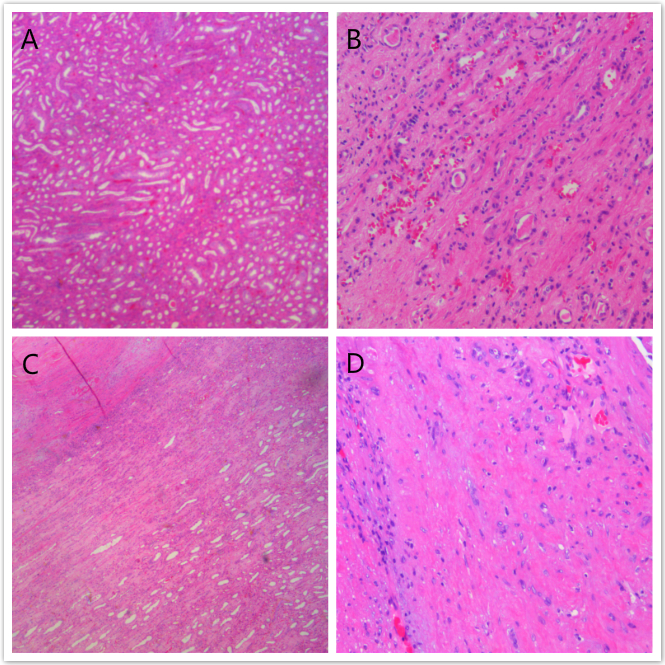


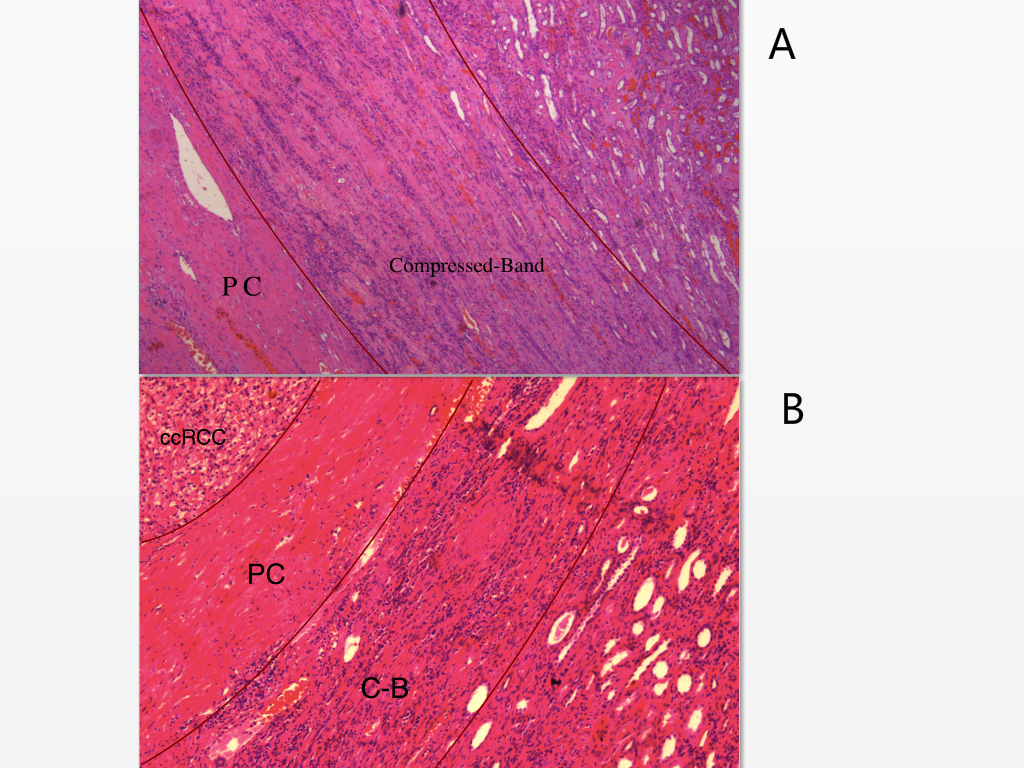


Supplementary Figure legends

Fig 1 (A-D): Chronic inflammation (CI) in non-neoplastic parenchyma by using random objective microscopic fields: A -(40x), grade 0; B- (40x), grade 1; C- (40x), grade 2; D (40x), grade 3.

Fig 2 (A-D): Glomerulosclerosis (GS) in non-neoplastic parenchyma: A -(40x), a single GS change; B- (10x), grade 1; C- (10x), grade 2; D -(10x), grade 3.

Fig 3 (A-D): Arteriosclerosis (AS) in non-neoplastic parenchyma: A- (40x), grade 0; B -(40x), grade 1; C- (40x), grade 2; D- (20x), grade 3.

Fig 4 (A-D): Nephrosclerosis(AS) in non-neoplastic parenchyma. A- (20x), grade 0; B- (20x), grade 1; C- (4x), grade 2; D- (4x), grade 3.

Fig 5(AB): peri-tumoral parenchyma (1-5 mm) consist of tumor, pseudo-capsule(PC), compressed band(CB) and normal parenchyma .
